# Bifunctional enzyme ATIC promotes propagation of hepatocellular carcinoma by regulating AMPK-mTOR-S6 K1 signaling

**DOI:** 10.1186/s12964-017-0208-8

**Published:** 2017-12-16

**Authors:** Minjing Li, Changzhu Jin, Maolei Xu, Ling Zhou, Defang Li, Yancun Yin

**Affiliations:** 1 Medicine and Pharmacy Research Center, Binzhou Medical University, No. 346 Guanhai Road, Yantai City, Shandong Province 264003 China; 2 Taishan Scholar Immunology Program, School of Basic Medical Sciences, Binzhou Medical University, No. 346 Guanhai Road, Yantai City, Shandong Province 264003 China; 3 The Key Laboratory of Traditional Chinese Medicine Prescription Effect and Clinical Evaluation of State Administration of Traditional Chinese Medicine, School of Pharmacy, Binzhou Medical University, No. 346 Guanhai Road, Yantai City, Shandong Province 264003 China; 4 Binzhou Medical University, No. 346 Guanhai Road, Yantai City, Shandong Province 264003 China

**Keywords:** ATIC, Hepatocellular carcinoma, AMPK

## Abstract

**Background:**

Hepatocellular carcinoma (HCC) is one of the cancer types with poor prognosis. To effectively treat HCC, new molecular targets and therapeutic approaches must be identified. 5-aminoimidazole-4-carboxamide ribonucleotide formyltransferase/inosine monophosphate (IMP) cyclohydrolase (ATIC), a bifunctional protein enzyme, catalyzes the last two steps of the de novo purine biosynthetic pathway. Whether ATIC contributes to cancer development remains unclear.

**Methods:**

ATIC mRNA levels in different types of human HCC samples or normal tissues were determined from Gene Expression across Normal and Tumor tissue (GENT) database. The expression level of ATIC in human HCC samples or cell lines were examined by RT-PCR and western blot. Overall survival and disease-free survival of HCC patients in the ATIC low and ATIC high groups were determined by Kaplan-Meier analysis. Effects of ATIC knockdown by lentivirus infection were evaluated on cell-proliferation, cell-apoptosis, colony formation and migration. The mechanisms involved in HCC cells growth, apoptosis and migration were analyzed by western blot and Compound C (C-C) rescue assays.

**Results:**

Here, we first demonstrated that expression of ATIC is aberrantly up-regulated in HCC tissues and high level of ATIC is correlated with poor survival in HCC patients. Knockdown of ATIC expression resulted in a dramatic decrease in proliferation, colony formation and migration of HCC cells. We also identified ATIC as a novel regulator of adenosine monophosphate-activated protein kinase (AMPK) and its downstream signaling mammalian target of rapamycin (mTOR). ATIC suppresses AMPK activation, thus activates mTOR-S6 K1-S6 signaling and supports growth and motility activity of HCC cells.

**Conclusion:**

Taken together, our results indicate that ATIC acts as an oncogenic gene that promotes survival, proliferation and migration by targeting AMPK-mTOR-S6 K1 signaling.

**Electronic supplementary material:**

The online version of this article (10.1186/s12964-017-0208-8) contains supplementary material, which is available to authorized users.

## Background

Hepatocellular Carcinoma (HCC), one of the most common and lethal cancers, is the second leading cause of cancer death worldwide [1]. An estimated 782,500 new liver cancer cases and 745,500 deaths occurred worldwide during 2012. Furthermore, incidence of HCC is increasing in areas with historically low rates, such as Western Europe and Northern America. The clinical outcome of HCC remains poor, mainly because the recurrence rates are high even after surgical resection; tumor recurrence complicates more than 70% of cases at five years after resection [2]. Seriously, effective pharmacologic agents have not yet been developed for the treatment of HCC [3]. Although many signaling pathways have been implicated in HCC, the molecular basis of HCC initiation and development remains largely unknown [3]. Therefore, the identification of the molecular mechanisms underlying the development and progression of HCC is still essential.

One potential important hallmark of cancer is metabolic reprogramming. To satisfy the metabolic requirements associated with a high growth rate, cancer cells often increase the use of anabolic pathways. Cancer cells maintain de novo purine synthesis pathway at high levels that support anabolic growth [4, 5]. The de novo purine biosynthetic pathway is a highly conserved, energy-intensive pathway that generates inositol monophosphate (IMP) from phosphoribosyl pyrophosphate (PRPP). The de novo purine synthesis pathway contains 10 sequential steps, beginning with phosphoribosyl pyrophosphate and ending with IMP. ATIC, is the last enzyme in this pathway and is a cytosolic enzyme that includes a transformylase domain, which transfers a formyl group to AICAR to produce the intermediate formyl-AICAR (FAICAR) and IMP [6]. Purine synthesis pathway is strongly correlated with rates of proliferation of cancer cells [7, 8]. However, whether and how ATIC modulates progression of cancer remains largely unknown.

In the present study, our data suggest that ATIC is overexpressed in HCC and the high level of ATIC is correlated with poor prognosis of HCC patients. In addition, ATIC promotes the proliferation and migration of HepG2 cells. Inhibition of ATIC expression significantly decreased the proliferation and migration ability of HepG2 cells through regulating the AMPK-mTOR-S6 K1 signaling axis. This finding suggests that targeting ATIC may effectively treat HCC.

## Methods

### Reagents and antibodies

The biochemical reagent 5-Aminoimidazole-4-carboxamide-1-β-D-ribofuranoside (AICAR) and Compound C (C-C) was purchased from Sigma Chemical (St Louis, MO). The following antibodies were used: anti-p-AMPKα (T172), p-mTOR (S2448), p-p70S6K(T389) and p-S6(S235/S236) (Cell Signaling Technology, Beverly, MA); ATIC (10726–1-AP), AMPKα (10929–2-AP), mTOR (20657–1-AP), S6 K1 (14485–1-AP), S6 (14823–1-AP), Caspase 3 (66470–1-Ig) and GAPDH (60004–1-Ig) (Proteintech Group, Chicago, IL).

### Cell culture and tissues samples

All the cell lines were obtained from Cell Lines Bank, Chinese Academy of Science (Shanghai, China). The cells were maintained in DMEM containing 10% fetal bovine serum, 100 U/ml of penicillin and 100 mg/ml streptomycin sulfate and incubated at 37 °C in a humidified atmosphere of 5% CO_2_. The 12 pairs of HCC and normal liver tissues were collected from 12 HCC patients who underwent surgery at Affiliated Hospital of Binzhou Medical University during the period from 2007 to 2014. The diagnosis of HCC was confirmed by biopsy. No radiotherapy and chemotherapy were conducted before the operation. The Ethical Committee of Binzhou Medical University approved the tissue collection and studies with collected tumor or normal tissues. In addition, all patients gave written informed consent.

### Gene expression and survival analysis

ATIC mRNA levels in different types of human HCC samples or normal tissues were determined from Gene Expression across Normal and Tumor tissue (GENT) database as described [9] (http://medicalgenome.kribb.re.kr/GENT/index.php). For survival analysis, we analyzed publicly available gene expression datasets from human cancer studies as described [10]. Data was obtained from the Liver Hepatocellular Carcinoma TCGA database, total 442 samples (http://www.cbioportal.org/). Some samples with incomplete data were discarded. Patients were separated into two groups based on whether they had higher or lower than the median expression level of ATIC to perform Kaplan-Meier survival analysis.

### Quantitative real-time PCR

Cells or tissues were lysed, total RNAs were obtained using Trizol Reagent (Invitrogen, Carlsbad, CA). cDNAs were prepared with random hexamers from mRNA, using the AMV reverse-transcription kit (Promega Biotechnology, Madison, WI). ATIC and reference GAPDH was amplified by real-time PCR using the Power SYBR Green PCR master amplification mix (Life Technologies, Carlsbad, CA) and 320 nmol/l primers. The primer sequences for human ATIC were F: 5’-CACGCTCGAGTGACAGTG-3′; R: 5’-TCGGAGCTCTGCATCTCCG-3′. The primer sequences for human GAPDH were F: 5’-AACTTTGGCATTGTGGAAGGA-3′; R: 5’-AACATCATCCCTGCTTCCAC-3′. Quantitative PCR reactions were performed on the iQ5TM (Bio-Rad, Hercules, CA) for 35 cycles using a recommendatory annealing temperature (60 °C). Relative quantification with the comparative threshold cycle (Ct) was done using the Ct method.

### Lentivirus construction and infection

The GFP tagged lentiviral vector PLL3.7 was used to express shRNAs designed to target ATIC. The oligonucleotide sequences of the shRNAs target ATIC and AMPK used are: ATIC shRNA:

shRNA1: 5’-CTGGAATCCTAGCTCGTAA-3′; shRNA2: 5’-CTCTGAGTTGACGGGATTT-3′;

shRNA3: 5’-TGCAAAAGCTCTCAGGGAT-3′; shRNA4: 5’-CTGGAATCCTAGCTCGTAA-3′.

AMPK shRNA: 5’-CCTGGAAGTCACACAATAGAA-3’ [11]. For lentiviral supernatant production, HEK-293 T cells were grown on 10-cm culture plates to ~80% confluence. 45 μL PolyJet reagent was diluted in 0.5 mL of DMEM medium (serum and antibiotic free) and incubated at room temperature for 5 min. In addition, 5.5 μg pMD2G, 2 μg PsPAX2 and 7.5 μg of the shRNA lentiviral construct were dissolved in 0.5 mL of DMEM medium (serum and antibiotic free), and mixed with the pre-diluted PolyJet. The mixture solution was incubated for 20 min before addition to HEK-293 T cells. After 5 h, the supernatant was discarded and replaced with 10 mL of DMEM with 10% FBS without antibiotics. Supernatant containing lentivirus was harvested 48 h and 72 h later. After filtering, lentivirus was used for the following infection. For infection, HCC cells were incubated with the lentivirus supernatant for 6 h. Culture medium was discarded, and cells were cultured in complete DMEM medium with 10% FBS for 24 h. This procedure was repeated for a second infection. GFP positive shRNA-encoding lentivirus-infected cells were sorted by flow cytometry 2 days after second infection.

### Western blot

Cells or tissue samples were lysed with ice-cold RIPA lysis buffer containing protease inhibitors (PMSF, Aprotinin and phosSTOP). Lysate was incubated on ice for 30 min and then centrifuged for 20 min at 12,000 g to remove debris. Proteins were boiled in 1× loading buffer for 10 min, protein samples were resolved by SDS-PAGE and proteins were transferred electrophoretically to PVDF membrane (250 mA, 90 min). Membranes were incubated with primary antibodies overnight at 4 °C and appropriate HRP-secondary antibodies for 1 h at room temperature. Detection was performed with chemiluminescent agents. Images were gathered by Alpha Innotech’s FluorChem imaging system. Densitometric analysis of blots was performed with Image J. All the experiments were repeated 3 times. A representative experiment is shown.

### In vitro kinase assays

The in vitro kinase assay was performed essentially as we described [12]. Recombinant S6 K1 protein (#40062, BPS Bioscience, San Diego, CA) and immunoprecipitated mTOR proteins were used for kinase assays. For immunoprecipitation of mTOR proteins, HepG2 cells were infected with scramble or ATIC shRNA for 48 h then treated with or without 10 μM C-C for 24 h. Then cells were harvested in lysis buffer respectively (0.3% CHAPS, 40 mM Hepes pH 7.5, 120 mM NaCl, 1 mM EDTA, 10 mM pyrophosphate, 10 mM glycerophosphate, 50 mM NaF, 0.5 mM orthovanadate). One mg of total proteins was immunoprecipitated with 3 μg of mTOR antibodies for 90 mins at 4 °C. Target proteins were collected by incubation with protein G Sepharose beads for 60 mins at 4 °C. To remove low-affinity binding contaminants, the beads-proteins complexes were extensively washed with cold high salt buffer (20 mM Hepes, pH 7.5, 2 M NaCl), followed by washing three times with cold lysis buffer and once with cold mTOR kinase buffer (25 mM Hepes pH 7.4, 50 mM KCl, 10 mM MgCl2). Then the beads-proteins complexes and 50 ng S6 K1 proteins were incubated in a final volume of 90 μl at 37 °C in the mTOR kinase buffer containing 500 μM ATP. 30 mins later, the reaction was stopped with the addition of 30 μl 4 × loading buffer and boiled for 10 min. The phosphorylation of S6 K1 was analyzed by western blot.

### Direct ELISA for intracellular AICAR

Intracellular AICAR level of the HCC tissue or cell line was measured by the human AICART ELISA kit (#E-EL-H1467c, ElabScience, Wuhan, China). Briefly, the cells or the chopped HCC tissues were rinsed in ice-cold PBS to remove excess medium or blood thoroughly. Then repeat the freeze-thaw process for several times until the tissue or cell samples are lysed fully. The homogenates are then centrifuged to get the supernatant. Added standard media or sample supernatant to the Micro ELISA Plate. Incubate the plate for 90 min at 37 °C. After washing three times with PBS, nonspecific binding was blocked in 1.0% bovine serum albumin. Add 1 Ab. The wells were then incubated with the biotinylated primary antibody and the HRP conjugated streptavidin. The TMB Substrate Reagent was added followed by quenching with stop solution and an absorbance reading at 450 nm. All values were in the linear range and were normalized for cell number or tissue weight.

### Cell growth/proliferation/apoptosis assay

Cells were transfected with shRNA targeting ATIC or a scrambled shRNA (shScr), then cell growth/proliferation/apoptosis were tested. For cell growth assay, cells were seeded into 12-well plates (8000/well) after 24 h transfected. Cell growth was evaluated by calculating the cell number with TC20™ automated cell counter (Bio-Rad, Hercules, CA) at different time points. The EdU Kit (#C10310–1, Ribobio, Guangzhou, China) was used to test the proliferation of the HCC cells as we described previously [12]. For cell apoptosis assay, cells were collected after 48 h transfected. Then cells were staining by Annexin V-PE/7-AAD apoptosis detection kit (#559783, BD Bioscience, San Jose, CA) as we described previously [13]. Fluorescence signals from at least 10,000 cells were collected by FACS (Beckman, Fullerton, CA) to determine the percentage of apoptotic cells.

### Colony formation assay

HepG2 cells were transfected with shRNA targeting ATIC or a scrambled shRNA. After 24 h, cells were plated in 12-well plates at 1500 cells/well. Then cells were allowed to grow in the absence of shRNA for 10 days to form colonies. The cells were fixed and stained with Giemsa dye liquor to visualize cell colonies. Images were gathered by light microscope. The number and size of colony was measured by NIS-Elements F3.0 imaging system (Bio-Rad, Hercules, CA). The relative number and size of colonies in six random visual fields (40X) under light microscope is shown.

### Wound healing assay

HCC cells were transfected with indicated shRNAs. When cells reached 90% confluence, a 1 mm scratch was placed through the middle of the confluent cultures with a pipette tip. The area of scratch was recorded by taking images under a phase-contrast microscope every 6 h. The gap width was measured by NIS-Elements F3.0 imaging system (Bio-Rad, Hercules, CA).

### Transwell assay

Cell migration was determined using transwell insert chambers. HCC cells were transfected with ATIC shRNA or scrambled shRNA. 24 h later, cells were harvested and 3 × 10^4^ cells were added to the upper chamber in serum-free medium. Then 750 μL of chemoattractant (medium supplemented with 10% FBS) was added to the basal chambers. After incubating for 8 h, migrated cells were fixed with 20% methanol, stained with 0.1% crystal violet. Cells beneath the upper chambers were counted microscopically.

### Statistical analysis

All data are presented as the means ± SD. Data were compared using two-tailed Student’s t-test. The survival between two groups was analyzed using log-rank test. The correlation of ATIC mRNA expression with clinicopathologic features was analyzed using chi-square test. Differences were defined as statistically significant if *p* < 0.05.

## Results

### ATIC is up-regulated in HCC tissues and cell lines

**Fig. 1:**
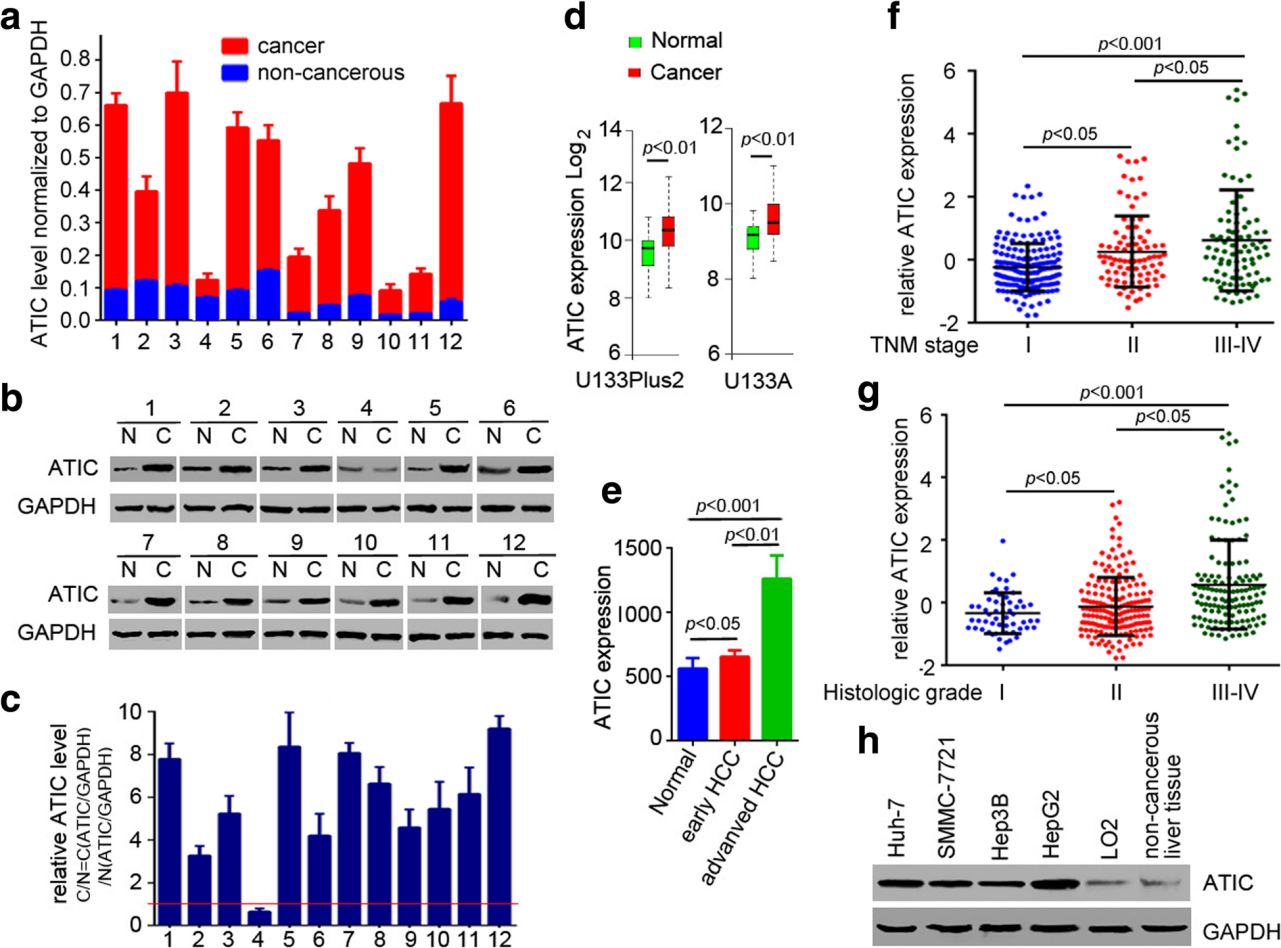
ATIC is up-regulated in HCC patients. **a,** RT-PCR analysis shows the mRNA level of ATIC in 12 pairs of HCC cancers and the adjacent non-cancerous liver tissues. Overexpression of ATIC was observed in 11 out of 12 HCC patient samples. ATIC mRNA expression level in HCCs and non-cancerous tissues were normalized to GAPDH. Experiments were repeated three times, Values represent mean ± SD. **b,** the protein level of ATIC was analyzed by Western blot in 12 representative pairs of HCC tumors and the adjacent non-cancerous liver tissues. A representative of three experiments is shown. N, Non-cancerous; C, Cancer. **c,** the relative level of ATIC protein was quantified using Image J. Fold change of ATIC protein with respect to non-cancerous specimens was normalized to GAPDH. Values represent mean ± SD, *N* = 3. **d,** Log2-transformed fold changes of ATIC mRNA expression level in liver normal and tumor tissues. Data from GENT database. (mean ± S.D., Unpaired t-test; U133plus2 data set, Cancer *N* = 182, Normal *N* = 25; U133A data set, Cancer *N* = 156, Normal *N* = 52.) **e,** ATIC mRNA levels in indicated samples. Data from GENT U133Plus2 data set. Normal *N* = 8, early HCC N = 18, advanced HCC *N* = 17. **f,** ATIC mRNA levels in HCC samples with different TNM stage. TNM Stage I *N* = 172, II *N* = 86, III-IV *N* = 90. Data from TCGA database. **g,** ATIC mRNA levels in HCC samples with different histologic grade. Histologic grade I *N* = 55, II *N* = 178, III-IV *N* = 134. Data from TCGA database. **h,** Expression of ATIC in indicated cell lines and adjacent non-cancerous liver tissue from patient #1 was analyzed by western blot

To explore the potential role of ATIC in HCC, we first examined the expression level of ATIC in a small cohort of human HCC samples with their corresponding non-cancerous tissues by RT-PCR and western blot. ATTC expression was significantly elevated in 11 of 12 (91.67%) cases compared to adjacent non-cancerous tissues (Fig. 1a). ATIC was also up-regulated in these HCC samples as assessed by western blotting (Fig. 1b and c). Consistent with these results, GENT analysis results showed that ATIC expression was significantly up-regulated in HCC samples compared to normal tissues (Fig. 1d). In addition, ATIC was more elevated in advanced HCC (Fig. 1e). We next performed an *in-silico* analysis of the expression level of ATIC using data from TCGA. Concordantly, the expression of ATIC significantly increased with HCC progression from TNM stage I to IV (Fig. 1f). Also, the expression of ATIC was elevated along with HCC progression of histologic grade (Fig. 1g). We further examined the expression of ATIC in several HCC cell lines, including Huh-7, SMMC-7721, Hep3B and HepG2. Western blot results showed that ATIC protein was abundantly expressed in HCC. Together, these results indicate that ATIC is highly expressed by HCC cells and may support HCC development.

### Up-regulated expression of ATIC is correlated with HCC progression

To determine whether the ATIC upregulation found in HCC tissues and cell lines was related to clinical indicators, the association between ATIC expression level and the clinical pathological characteristics of HCC patients was analyzed (Table 1). ATIC expression was markedly associated with serum AFP level (*p* = 0.0011), hepatitis status (*p* = 0.0352), vascular invasion (*p* = 0.0384), histologic grade (*p* = 0.0006) and Tumor node metastasis (TNM) stage (*p* = 0.0086) in HCC patients.

**Table 1:** Correlation of ATIC mRNA expression with clinicopathologic features in HCC

	ATIC mRNA expression^△^		*p* value
Variable	Low (117)	high (117)	(chi-square)
Gender			
Male	83	81	0.8865
Female	34	36	
Diagnosis Age			
≤ 50	23	30	0.3488
> 50	94	87	
AFP (ng/mL)			
≤ 200	97	74	0.0011*
> 200	20	43	
Hepatitis status			
Negative	61	44	0.0352*
Positive	56	73	
Vascular Invasion			
No	85	69	0.0384*
Yes	32	48	
Histologic Grade			
G1	17	9	0.0006*
G2	64	43	
G3-G4	36	65	
TNM stage			
I	78	56	0.0086*
II	24	31	
III-IV	15	30	

△, A median split is performed. *, Represents *p* values with significant difference

TNM, Tumor node metastasis. Data from TCGA database (http://www.cbioportal.org/)

**Fig. 2:**
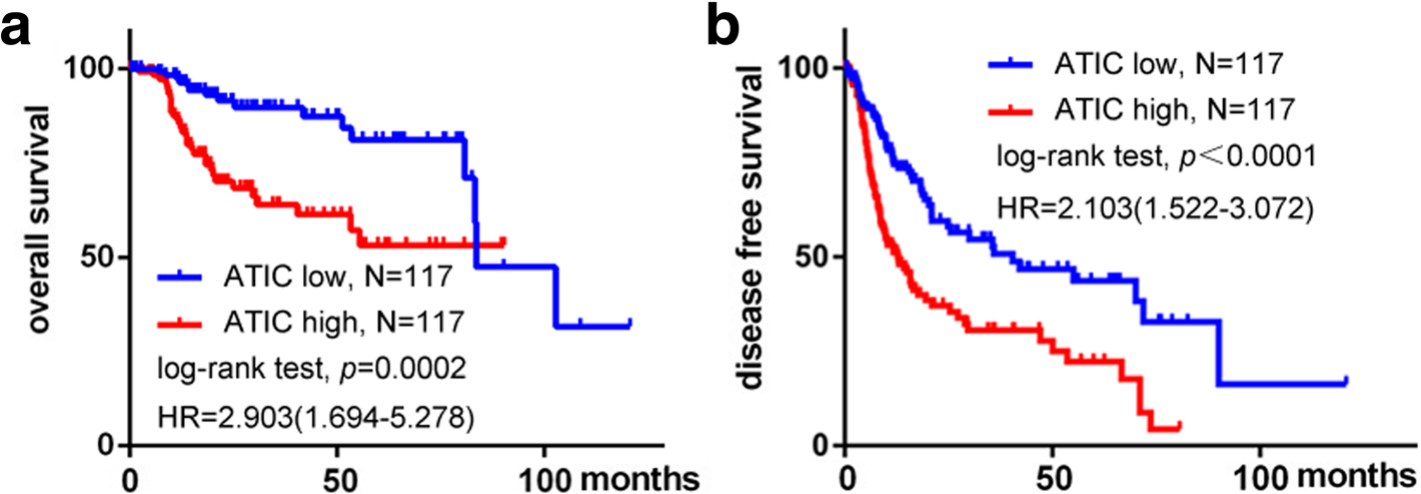
ATIC expression negatively correlates with survival of HCC patients. ATIC mRNA expression data from the Liver Hepatocellular Carcinoma TCGA database (http://www.cbioportal.org/) were normalized to total mRNA expression. Patients were separated into two groups based on whether expression of ATIC was higher or lower than the average expression levels, and % overall survival (**a**) or disease-free survival (**b**) vs. time was plotted

To elucidate the association of ATIC expression with clinical outcomes in HCC patients, we performed the Kaplan-Meier analysis of the relationship between ATIC expression and clinical endpoints of HCC patients. In HCC patients, high ATIC expression was significantly associated with shortened overall survival (Fig. 2a) as well as reduced disease-free survival (Fig. 2b). In addition, high TNM stage and histologic grade was significantly associated with poorer clinical outcomes (Sup. Fig. 1). These results suggest that ATIC may support propagation of HCC and appears to be a strong marker of poor prognosis of HCC patients.

### ATIC knockdown suppresses HCC cell motility activity

**Fig. 3:**
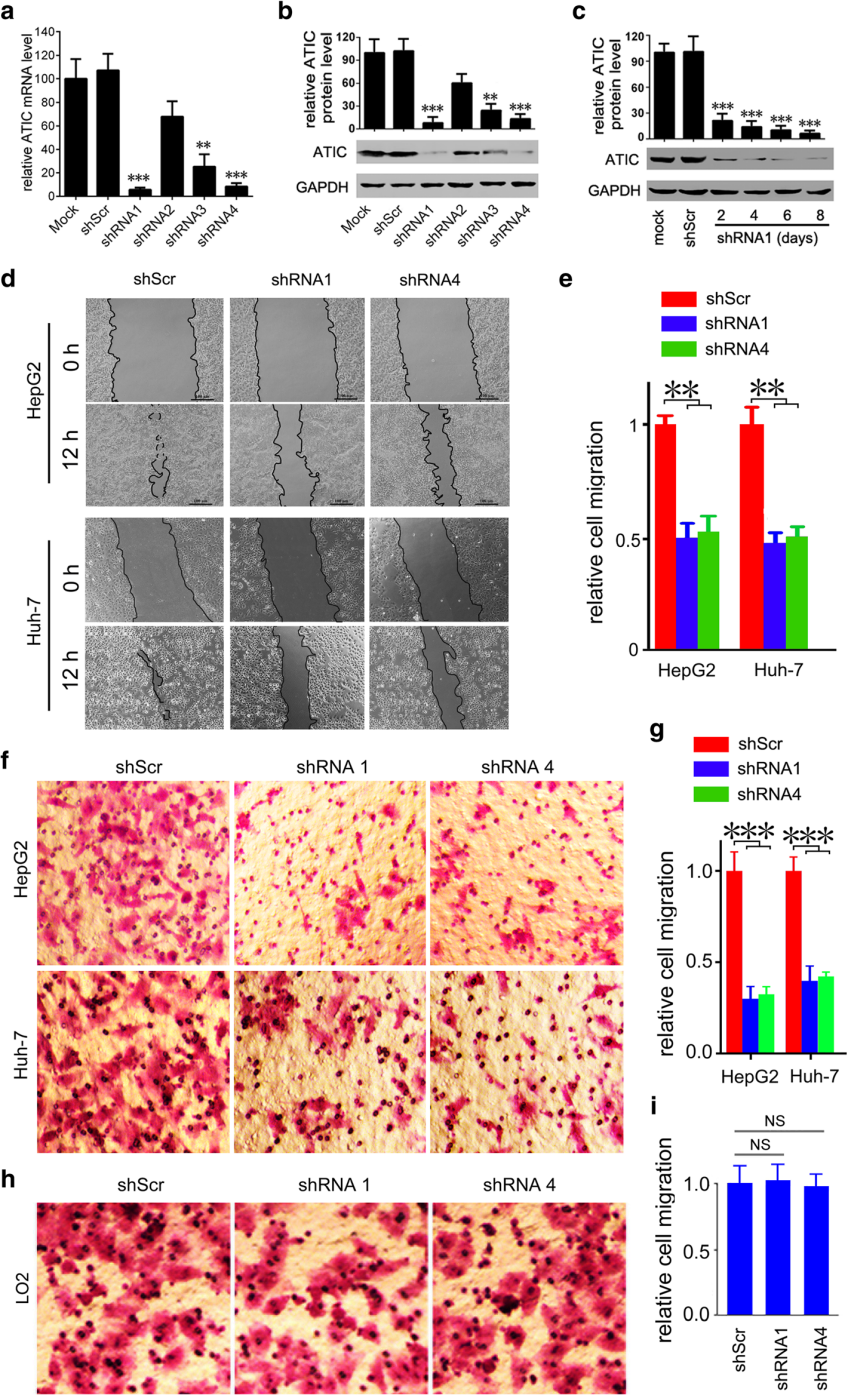
Knockdown of ATIC expression suppresses migration of HCC cells. **a,** HepG2 cells were transfected with indicated shRNA for 24 h followed by real-time-PCR analysis of ATIC transcription. *N* = 3, **, *p* < 0.005, ***, *p* < 0.001, compared to shScr and mock group. **b,** HepG2 cells were transfected with indicated shRNA for 72 h followed by western blot analysis of ATIC expression. The result of densitometric analysis was plotted after normalization to GAPDH. N = 3, **, p < 0.005, ***, p < 0.001, compared to shScr and mock group. **c,** HepG2 cells were transfected with shRNA1 for indicated times followed by western blot analysis of ATIC expression. N = 3, ***, p < 0.001, compared to shScr and mock group. **d,** Representative images of wound healing assay for HCC cells transfected with indicated shRNAs. The gap width at multiple constant points was measured and recorded at 12 h. Bar, 100 μm. **e,** Relative migration distance of cells was plotted. Migration distance in shScr group was set as 1. *N* = 4, **, *p* < 0.005. **f,** Representative images of transwell analyses to evaluate the migration ability of HCC cells transfected with indicated shRNAs. **g,** Relative migrated cell numbers were plotted after normalized to total number. N = 4, ***, *p* < 0.001. **h,** Representative images of transwell analyses to evaluate the migration ability of LO2 cells transfected with indicated shRNAs. **i,** Relative migrated cell numbers were plotted after normalized to total number. *N* = 4, NS, no significant

To further investigate the biological function of ATIC, we transiently depleted ATIC expression in HCC cells using shRNAs. The efficiency of the designed shRNAs was determined by evaluating the expression of ATIC in mRNA and protein levels in HCC cells. RT-PCR result showed that shRNAs 1, 3 and 4 could efficiently inhibit expression of ATIC in mRNA level compared to mock or shScr. (Fig. 3a). Specifically, the mRNA level of ATIC was decreased to <15% of control by shRNA1 and shRNA4 in HepG2 cells (Fig. 3a). Consistently, in protein level the shRNA showed similar knockdown efficiency (Fig. 3b). In addition, the shRNAs showed similar knockdown efficiency in Huh-7 (Additional file 1: Fig. S2A, B). Furthermore, we evaluated time-dependent knockdown efficiency of the shRNA. The western blot result suggested that the shRNAs were sufficient to maintain ATIC at low level for 8 days (Fig. 3c). Here, we selected shRNA1 and shRNA4 that showed greater knockdown efficiencies for next functional study. To investigate the possible role of ATIC in migration of HCC cells, we determined the migration ability of HepG2 and Huh-7 cells after knockdown expression of ATIC with shRNAs. ATIC-deficient cells migrated more slowly than cells treated with shScr in a wound healing assay (Fig. 3d, e). Then the role of ATIC in HCC cell migration was confirmed by the trans-well assay. Inhibiting expression of ATIC with either shRNA1 or shRNA4 led to a dramatic decrease in migration of HepG2 and Huh-7 cells (Fig. 3f, g). However,knockdown expression of ATIC has no effect on migration of normal liver cells LO2 (Fig. 3h, i). The efficiency of the knockdown of ATIC was determined (Additional file 1: Fig. S2C). Together, these results suggest that ATIC supports cell motility activity of HCC.

### ATIC knockdown suppresses HCC cell growth

**Fig. 4:**
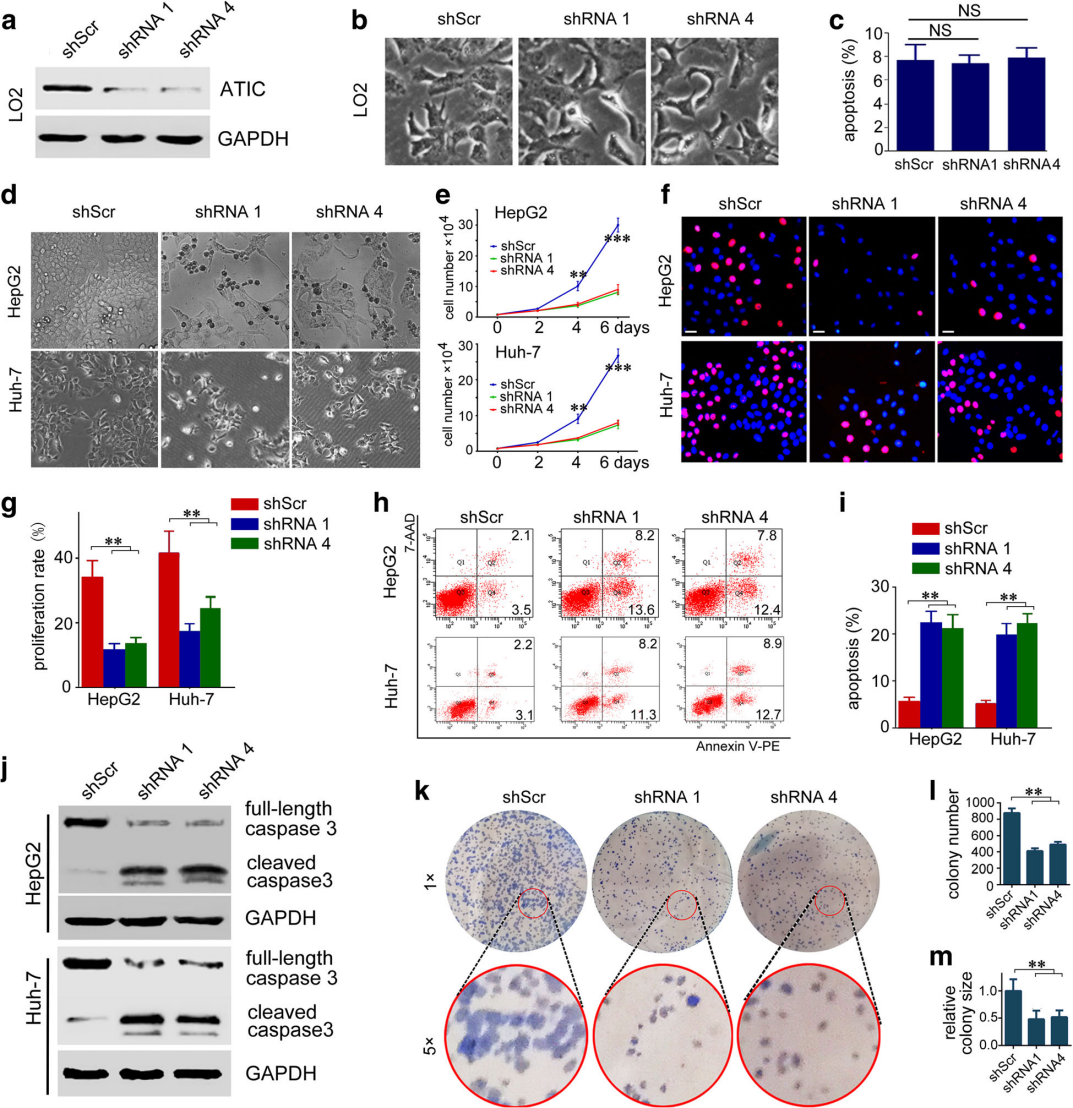
Knockdown of ATIC expression suppresses growth of HCC cells. **a,** LO2 cells were infected with indicated shRNA for 72 h. The efficiency of ATIC knockdown was determined by western blotting. **b,** Representative images of LO2 cells 5 days after transfection with indicated shRNAs. **c,** LO2 cells were infected with indicated shRNA for 72 h. Cell apoptosis are analyzed by flow cytometry. The apoptosis percentage was plotted. N = 3, NS, no significant. **d,** Representative images of HCC cells 5 days after transfection with indicated shRNAs. **e,** Cell numbers at indicated days after transfection with indicated shRNAs. *N* = 4, **, *p* < 0.005, ***, *p* < 0.001, compared to shRNA 1 and shRNA 4 groups. **f,** Cells were transfected with indicated shRNAs for 72 h followed by Hoechst and EdU labeling analysis proliferation. Representative images were shown. EdU-labeled (pink) indicated proliferation cells. Bars, 10 μm. **g,** the proliferation rate was plotted. *N* = 4, **, *p* < 0.005. **h,** Cells at 72 h post transfection with indicated shRNAs. Shown are representative flow cytometry plots. **i,** the apoptosis percentage was plotted. *N* = 3, **, *p* < 0.005. **j,** Cells were transfected with indicated shRNAs for 72 h, followed by western blot analysis of caspase 3 activation. **k,** Cells were transfected with indicated shRNAs. 24 h after transfection, 1500 cells were seeded in 12-well plates for 10 days followed by staining and quantification. Representative images were shown. **l,** colony number was plotted. *N* = 3, **, *p* < 0.005. **m,** relative colony size was plotted. The colony size in shScr group was set as 1. **, *p <* 0.005

Given that ATIC is an essential metabolic enzyme that performs critical functions in de novo purine biosynthesis, we first detect the biological function of ATIC in normal liver cells. Interestingly, we find that knockdown expression of ATIC has no effect on growth or apoptosis of normal hepatocyte cell LO2 (Fig. 4 a-c). To further explore the biological function of ATIC in HCC, HepG2 or Huh-7 cells were transiently transfected with shRNA1 or shRNA4 followed cell viability was measured by counting cell numbers. Transfection with shRNA1 or shRNA4 resulted in a dramatic decrease in HCC cell growth as well as visible cell death (Fig. 4d). Cell growth was much slower 4 days after transfection with ATIC shRNAs and was more apparent after 6 days (Fig. 4e). To explore the mechanisms underlying ATIC supports HCC cell growth, we compared the cell proliferation and apoptosis of HepG2 and Huh-7 cells treated with shScr or ATIC shRNAs. EdU labeling assays showed that knocking down expression of ATIC significantly decreased proliferation of HepG2 and Huh-7 cells (Fig. 4f, g and Additional file 1: Fig. S3). Furthermore, ATIC-deficient HCC cells had significantly increased levels of apoptosis compared to control cells treated with shScr (Fig. 4h, i). Conformably, ATIC-deficient HCC cells had significantly increased levels of cleaved caspase3 (Fig. 4j). These results indicated that both increased apoptosis and decreased proliferation might contribute to dramatic slowdown of cell growth after ATIC knockdown in HCC cells. We further tested the effect of ATIC knockdown on colony forming ability of HepG2 cells. There were fewer colonies formed in cells treated with shRNA1 or shRNA4 than that in cells treated with shScr (Fig. 4k, l). Meanwhile, the surviving colonies in cells treated with shRNA1 or shRNA4 tended to be much smaller than that in cells treated with shScr (Fig. 4k,m). This result, consistent with those of the cell proliferation and apoptosis analyses, indicates that ATIC is significant for HCC cell growth and survival.

### ATIC promotes AMPK- mTOR-S6 K1 signaling activation

**Fig. 5:**
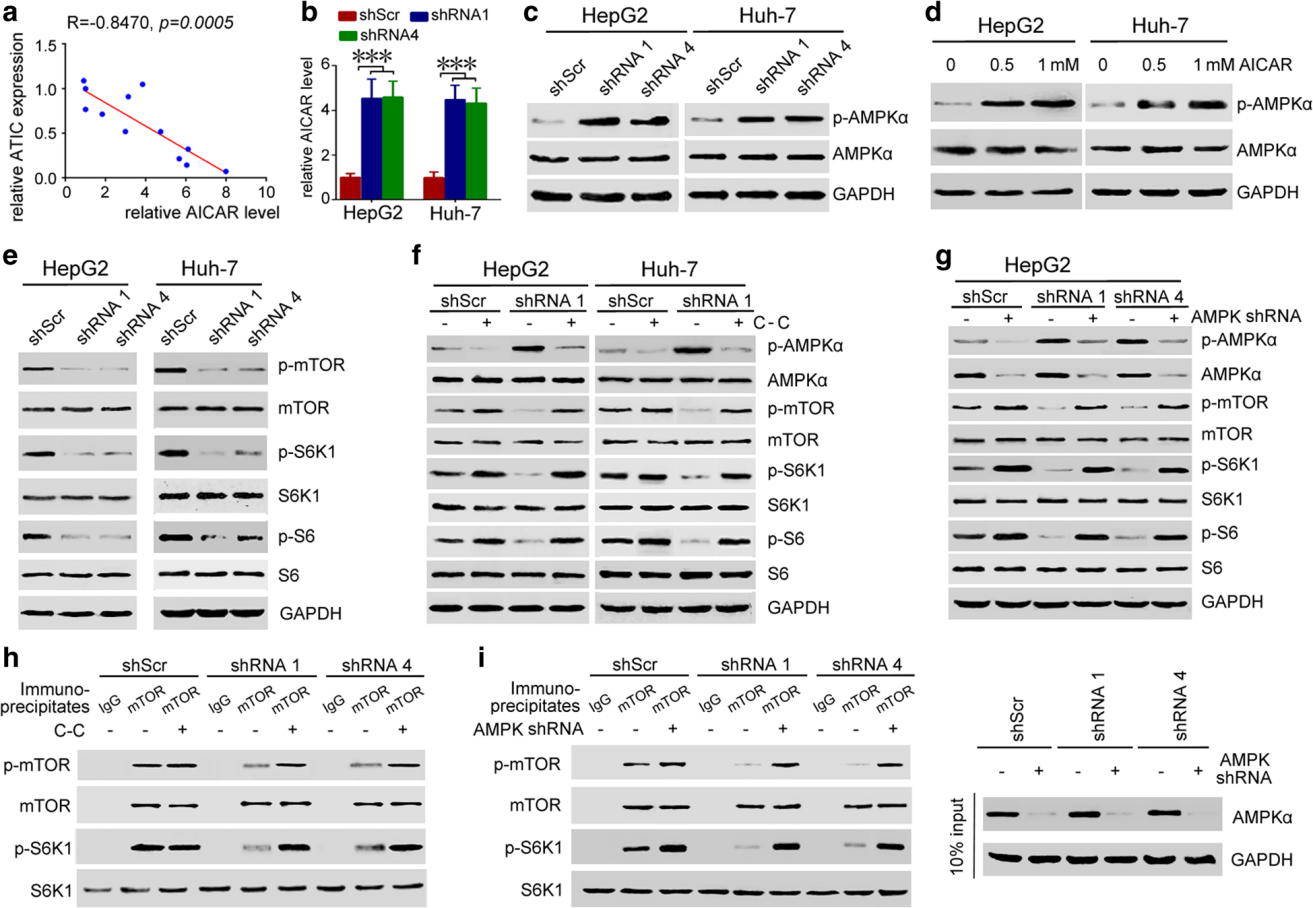
ATIC knockdown inhibits mTOR-S6 K1-S6 signaling through activating AMPK. **a,** Expression of ATIC was detected as in Fig. 1A. The AICAR level in 12 HCC cancer tissues was determined by ELISA assay. The expression of ATIC and AICAR level in patient #1 was set as 1 respectively. Correlation of relative expression of ATIC and AICAR level in 12 HCC patients was analyzed. **b,** cells were infected with indicated shRNAs for 72 h, followed by ELISA assays analysis AICAR level. The relative AICAR level was plotted. The AICAR level in shScr group was set as 1. ***, *p* < 0.001. **c-d,** HepG2 or Huh-7 cells were treated with indicated shRNAs (**c**) or AICAR (**d**) for 72 h. Status of AMPK was analyzed. **e,** cells were transfected with indicated shRNAs for 72 h. Status of mTOR-S6 K1-S6 signaling was analyzed. **f-g,** cells were transfected with indicated shRNA, 24 h later, cells were treated with 10 μM C-C (F) or AMPK shRNA (G) for 48 h. Status of AMPK-mTOR-S6 K1-S6 signaling was analyzed. **h-i,** HepG2 cells were infected with indicated shRNAs then treated with 10 μM C-C (**h**) or AMPK shRNA (**i**). mTOR were immunoprecipitated from these cells respectively. The immunoprecipitates were incubated with S6 K1 in a kinase assay system, followed by western blot analysis. (**i**, right panel), the efficiency of AMPK knockdown was determined by western blot

Previous studies have clearly showed that ATIC is a rate-limiting enzyme in the de novo purine synthesis pathway, and AICAR accumulates over time under conditions of lower ATIC expression [14, 15]. In addition, AICAR is an efficient activator of the energy sensor AMPK [16]. Hence, we hypothesized that AMPK and its downstream signaling may be involved in function of ATIC to support HCC cell growth and migration. As expected, our results indicate that lower levels of ATIC expression correlated with an increased intracellular level of AICAR of HCC patients (Fig. 5a). Knockdown expression of ATIC also induces intracellular accumulation of AICAR in HCC cells (Fig. 5b). In addition, treatment with shRNA1 or shRNA4 stimulated AMPK activation in HepG2 and Huh-7 cells (Fig. 5c). AICAR treatment also induced AMPK activation (Fig. 5d). Western blot results indicated that phosphorylation of mTOR, S6 K1 and S6 was markedly decreased in shRNA1 or shRNA4 treated cells compared to that in shScr treated cells (Fig. 5e). Furthermore, AMPK inhibitor C-C treatment rescued ATIC-deficient induced suppression of mTOR-S6 K1-S6 signaling (Fig. 5f). Silencing AMPK by shRNA also rescued ATIC-deficient induced inactivation of mTOR, S6 K1 and S6 in HepG2 cells (Fig. 5g). We next determined whether expression level of ATIC affect the kinase activity of endogenous mTOR by in vitro kinase assays. Compared to the immunoprecipitated mTOR from shScr treated cells, the immunoprecipitated mTOR from ATIC shRNAs-treated cells exhibited lower levels of serine phosphorylation and induced lower levels of S6 K1 phosphorylation (Fig. 5h, i). Furthermore, AMPK inhibitor C-C or AMPK shRNA treatment rescues ATIC-deficient induced kinase activity depression of mTOR (Fig. 5h, i). These results further support our finding that AMPK and mTOR play important roles in ATIC signaling. In summary, our results indicate that ATIC regulates a signaling network involving AMPK, mTOR, S6 K1 and S6 by regulating intracellular AICAR level in HCC cells.

### AMPK activation is involved in ATIC promoted growth and motility of HCC cells

**Fig. 6:**
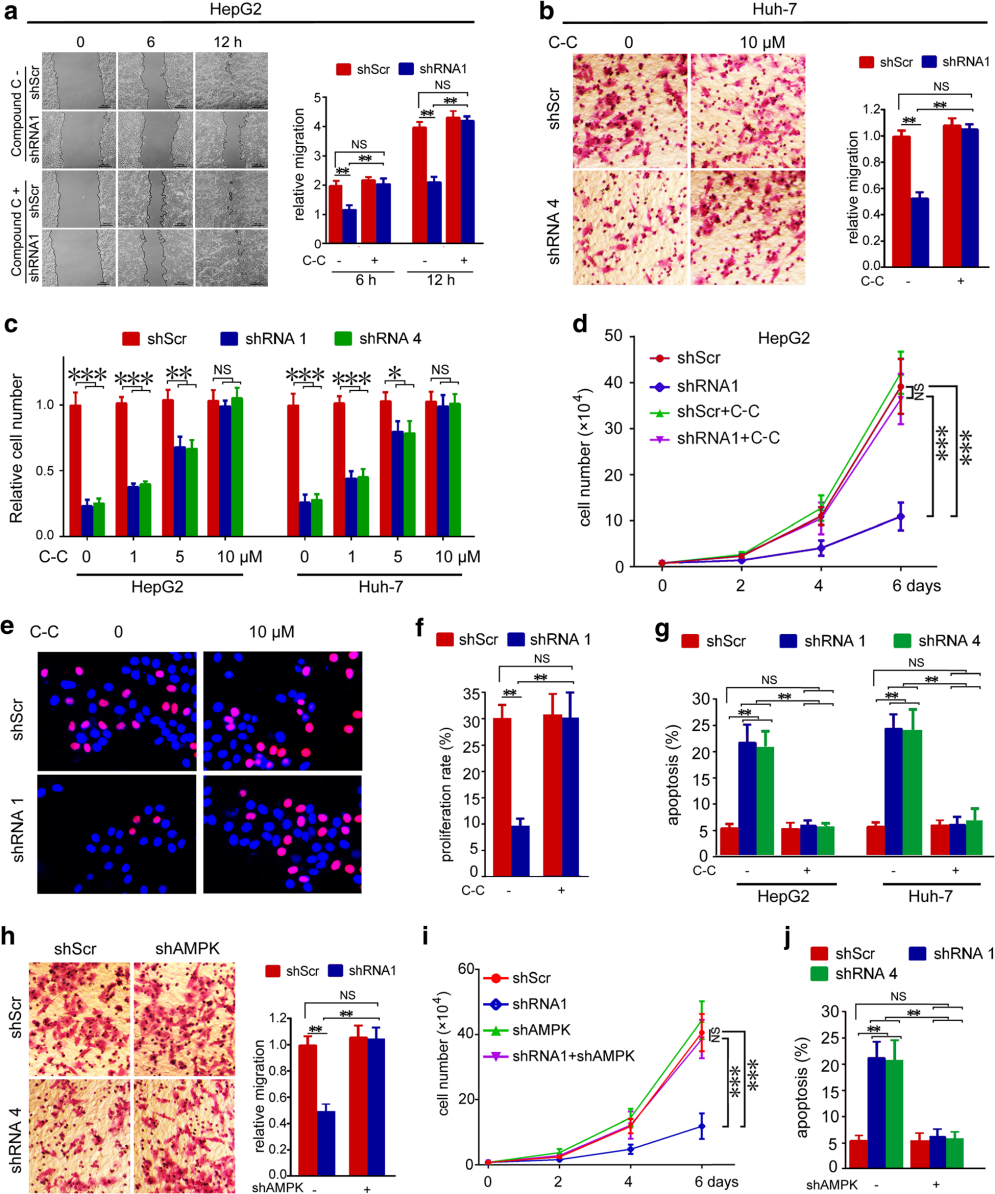
AMPK-mTOR-S6 K1-S6 signaling regulates growth and migration of HCC cells. **a-g,** Cells were transfected with indicated shRNA for 24 h, then cells were treated with or without C-C, followed by testing migration,growth,proliferation and apoptosis. (**a**) migration ability was analyzed by wound healing assay. Representative images (left) and relative migration distance (right) was plotted. Migration distance in shScr group at 6 h was set as 1. *N* = 4, **, *p* < 0.005, NS, no significant. (**b**) cell migration was analyzed by trans-well. Representative images (left) and relative migration cell numbers (right panel) were plotted. Migration cell numbers in shScr group was set as 1. N = 4, **, *p* < 0.005, NS, no significant. (**c**) Relative cell numbers at 4 days after transfection was plotted. N = 4, *, *p* < 0.05,**, *p* < 0.005,***, p < 0.001,NS, no significant. (**d**) cell numbers at indicated days after transfection was plotted. N = 4, ***, *p* < 0.001,NS, no significant. (**e**) cell proliferation was determined by Hoechst and EdU labeling assay. Representative images were shown. EdU-labeled (pink) indicated proliferation cells. (**f**) the proliferation rate was plotted. N = 4, **, *p* < 0.005, NS, no significant. (**g**) the apoptosis percentage was plotted. N = 3, **, *p* < 0.005, NS, no significant. **h-j,** HepG2 cells were infencted with ATIC shRNA for 24 h, then were infected with AMPK shRNA for 48 h. (**h**), cell migration was determined by trans-well assays. Representative images (left) and relative migration cell numbers (right) were plotted. Migration cell numbers in shScr group was set as 1. N = 4, **, *p* < 0.005, NS, no significant. (**i**), Cell numbers at indicated days after transfection was plotted. N = 4, ***, *p* < 0.001,NS, no significant. (**j**), Cell apoptosis was determined by flow cytometry. The apoptosis percentage was plotted. N = 3, **, *p* < 0.005, NS, no significant

Our previous data indicated that AMPK was markedly activated in ATIC-deficient cells. To determine whether AMPK is involved in ATIC induced migration of HCC cells, the above mentioned ATIC shRNA 1 or shScr was transfected into HepG2 cells followed by treating with or without AMPK inhibitor C-C. Wound-healing assay results showed that C-C treatment rescued ATIC-deficient induced migration inhibition (Fig. 6a). Trans-well assay showed similar results in Huh-7 cells with shRNA 4 (Fig. 6b). Next, cell growth was detected by counting cell numbers. Treatment with C-C rescued ATIC-deficient induced growth inhibition in a dose-dependent manner (Fig. 6c). In addition, 10 μM C-C treatment caused tumor cell growth similar to level of shScr transfected HepG2 cells further supporting that AMPK inhibition rescued ATIC deficiency (Fig. 6d). Furthermore, C-C treatment also rescued ATIC-deficient induced proliferation inhibition (Fig. 6e, f) and apoptosis (Fig. 6g). Similar to that observed from pharmacological inhibition of AMPK by C-C, knockdown expression of AMPK also rescued ATIC-deficient induced migration suppression (Fig. 6h), growth inhibition (Fig. 6i) and apoptosis (Fig. 6j) in HepG2 cells. We confirmed that the shRNA did knockdown expression of AMPK (Additional file 1: Fig. S4). Together, these assays indicate that the oncogenic function of ATIC is mediated by AMPK. In summary, our data suggest that ATIC supports HCC propagation by inhibition of AMPK activity and induction of mTOR-S6 K1-S6 signaling.

## Discussion

**Fig. 7:**
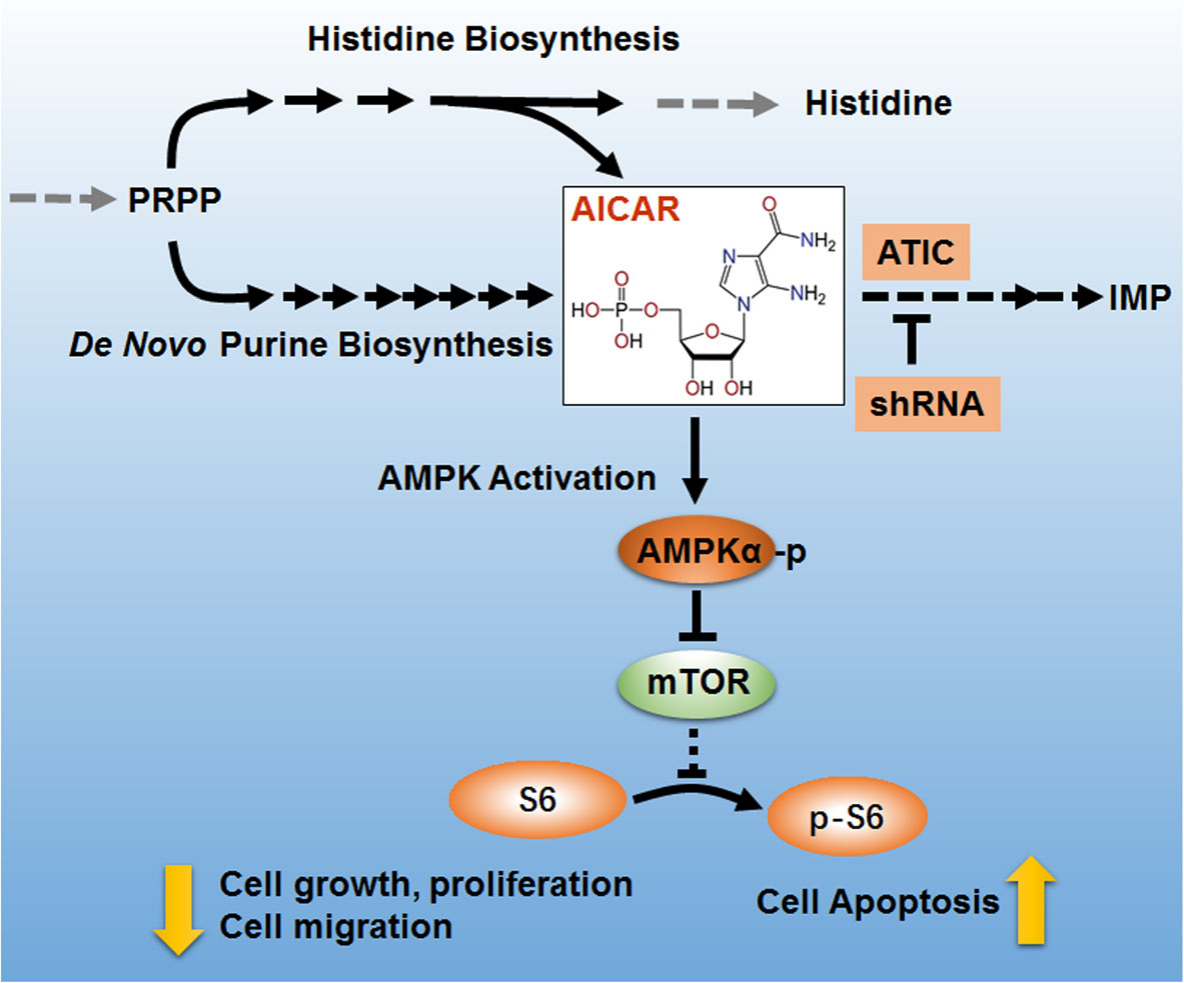
AMPK Activation by ATIC Knockdown. Inhibition of ATIC expression by shRNA leads to an increase in endogenous AICAR, which activates AMPK and inhibits its downstream pathway mTOR-S6 K1-S6. Finally, ATIC knockdown leads to decrease in cell proliferation and migration and increase in cell apoptosis.

The progression of cancer involves aberrant expression of oncogenes and tumor suppressor genes, as they deregulate multiple metabolic pathways and contribute to the neoplastic phenotype. Better understanding of specific metabolic checkpoints in cancer cells would allow the design of novel therapeutic strategies. Here, we first demonstrate knockdown expression of ATIC leads to an increase in endogenous AICAR, which regulates AMPK and its downstream mTOR-S6 K1-S6 pathway. Finally, inhibition of ATIC expression resulted in a dramatic decrease in proliferation, migration and increase in apoptosis (Fig. 7). The first identification of AMPK-mTOR axis as a novel target of ATIC may help to understand how ATIC drives tumor progression and contribute to design of novel therapeutic strategies for human HCC.

ATIC, the product of the *purH* gene, is a 64 kDa bifunctional enzyme that possesses the final two activities in de novo purine biosynthesis, AICAR transformylase (AICART) and IMP cyclohydrolase [17, 18]. Recent study demonstrates that inhibition AICART activity of ATIC and a subsequent rise in intracellular 5-Aminoimidazole-4-carboxamide ribonucleotide (ZMP) plays a significant role in the anti-tumorigenic effects of the drug pemetrexed, which is used against non-small cell lung cancer [19, 20]. Furthermore, the *ATIC* 347C > G polymorphism may influence the levels of adenosine after methotrexate treatment, which may affect the histologic response of osteosarcoma [21]. Despite these elegant observations, the significance of ATIC in human cancer has not been fully investigated. The present study strongly commended ATIC as a predictor for prognosis in HCC. Furthermore, our results suggested that ATIC may support HCC cells growth and migration by regulating AMPK-mTOR-S6 K1-S6 signaling. Overall, our findings might lead to a breakthrough in the field of ATIC function in supporting cancer cell growth or migration. Further study will be necessary to determine whether ATIC supports HCC development in vivo by using orthotopic xenograft models. And function of ATIC will need to be fully validated in clinical studies.

AMPK functions to monitor and maintain energy homeostasis at the cellular and organism level in response to metabolic stress. AMPK activation has been demonstrated to inhibit the proliferation of a variety of cancer cells, resulting in activation of p53, cell-cycle arrest and apoptosis depending on the cell line studied [22–24]. As the unique ability of AMPK to regulate cancer cell proliferation, there is a growing interest in the therapeutic exploitation of the AMPK pathway in cancer treatment. This premise is supported by preclinical findings that AMPK activators metformin and A-769662 exhibited efficacy in blocking carcinogen-induced tumorigenesis and suppressing tumor growth in vivo in different animal models [25, 26]. Recently Daniel J and colleagues showed that Cpd14, a selective inhibitor of ATIC homodimerization, leaded to an increase in intracellular ZMP. And endogenous ZMP activates AMPK and its downstream signaling pathways [27]. Consistent with previous studies, the current study first demonstrates that knockdown ATIC promotes AMPK activation and inhibits mTOR-S6 K1-S6 signaling in HCC cells. Our study demonstrates that ATIC may be a new molecular approach to selective AMPK activation. Further studies are needed to ascertain the mechanisms by which ATIC inhibition controls AMPK activation in vivo, but one possible route may be via the accumulation of AICAR observed in our cell assays.

In summary, we first demonstrated that the up-regulated expression of ATIC negatively correlates with the recurrence and overall survival of HCC patients. ATIC seems to be involved in HCC growth and migration through the AMPK and mTOR signaling pathways. Furthermore, purine levels in mammalian cells are maintained by a coordinated action of the salvage and de novo biosynthetic pathways. Previous studies have suggested that normal cells preferentially utilize salvage for the synthesis of purines while tumor cells favor the de novo pathway [28, 29]. Transformed cells depend on de novo nucleotide synthesis to support increased RNA production and DNA replication. Elevated nucleotide biosynthesis has been observed in many cancers. Thus the anti-ATIC strategy may effectively inhibit cancer growth yet with minimal toxicity to normal cells [30]. Therefore, targeting ATIC may hold promise as a novel strategy for human HCC treatment. It may be desirable to test the effect of ATIC inhibitor and AICAR for HCC treatment. It will also be interesting to study the potential different roles of ATIC in different types of cancer.

## Conclusions

In conclusion, our study demonstrates the potential tumor stimulative role of ATIC in HCC. Moreover, ATIC could, at least partially, affect the AMPK-mTOR-S6 K1 signaling pathway, which is important tumor-related signaling pathway in HCC, by regulating de novo purine synthesis pathway. Taken together, although more in-depth mechanisms and prognostic roles for ATIC in HCC need to be confirmed in the future, our findings provide a preliminary basis to explore ATIC as a potential molecular target for the development of HCC therapies.

## Additional file

Additional file 1: Supplementary Materials. (DOCX 818 kb)

## Abbreviations

AICAR: 5-Aminoimidazole-4-carboxamide-1-β-D-ribofuranoside
AMPK: adenosine monophosphate-activated protein kinase
ATIC: 5-aminoimidazole-4-carboxamide ribonucleotide formyltransferase/inosine monophosphate cyclohydrolase
C-C: Compound C
GENT: Gene Expression across Normal and Tumor tissue
HCC: Hepatocellular carcinoma
IMP: inositol monophosphate
mTOR: Mammalian target of rapamycin
PRPP: phosphoribosyl pyrophosphate
TNM: Tumor node metastasis
ZMP: 5-Aminoimidazole-4-carboxamide ribonucleotide

## References

[1] Torre LA, Bray F, Siegel RL, Ferlay J, Lortet-Tieulent J, Jemal A (2015). Global cancer statistics, 2012. Ca-Cancer J Clin.

[2] Poon RT (2011). Prevention of recurrence after resection of hepatocellular carcinoma: a daunting challenge. Hepatology.

[3] Dutta R, Mahato RI (2017). Recent advances in hepatocellular carcinoma therapy. Pharmacol Ther.

[4] Martinez-Outschoorn UE, Peiris-Pages M, Pestell RG, Sotgia F, Lisanti MP (2017). Cancer metabolism: a therapeutic perspective. Nat Rev Clin Oncol.

[5] Yamaoka T, Kondo M, Honda S (1997). Amidophosphoribosyltransferase limits the rate of cell growth-linked de novo purine biosynthesis in the presence of constant capacity of salvage purine biosynthesis. J Biol Chem.

[6] Beardsley GP, Rayl EA, Gunn K (1998). Structure and functional relationships in human pur H. Adv Exp Med Biol.

[7] Chan CY, Zhao H, Pugh RJ (2015). Purinosome formation as a function of the cell cycle. P Natl Acad SCI USA..

[8] Liu X, Chhipa RR, Pooya S (2014). Discrete mechanisms of mTOR and cell cycle regulation by AMPK agonists independent of AMPK. P Natl Acad SCI USA.

[9] Shin G, Kang TW, Yang S, Baek SJ, Jeong YS, Kim SYGENT (2011). Gene expression database of normal and tumor tissues. Cancer Inform.

[10] Cerami E, Gao J, Dogrusoz U (2012). The cBio cancer genomics portal: an open platform for exploring multidimensional cancer genomics data. Cancer Discov.

[11] Kim EK, Lim S, Park JM (2012). Human mesenchymal stem cell differentiation to the osteogenic or adipogenic lineage is regulated by AMP-activated protein kinase. J Cell Physiol.

[12] Yin Y, Hua H, Li M (2016). mTORC2 promotes type I insulin-like growth factor receptor and insulin receptor activation through the tyrosine kinase activity of mTOR. Cell Res.

[13] Chen C, Yin Y, Li C (2016). ACER3 supports development of acute myeloid leukemia. Biochem Bioph Res Co.

[14] Wang W, Fridman A, Blackledge W (2009). The phosphatidylinositol 3-kinase/akt cassette regulates purine nucleotide synthesis. J Biol Chem.

[15] Marie S, Heron B, Bitoun P, Timmerman T, Van Den Berghe G, Vincent MF (2004). AICA-ribosiduria: a novel, neurologically devastating inborn error of purine biosynthesis caused by mutation of ATIC. Am J Hum Genet.

[16] Corton JM, Gillespie JG, Hawley SA, Hardie DG (1995). 5-aminoimidazole-4-carboxamide ribonucleoside. A specific method for activating AMP-activated protein kinase in intact cells?. Eur J Biochem.

[17] Greasley SE, Horton P, Ramcharan J, Beardsley GP, Benkovic SJ, Wilson IA (2001). Crystal structure of a bifunctional transformylase and cyclohydrolase enzyme in purine biosynthesis. Nat Struct Biol.

[18] Vergis JM, Bulock KG, Fleming KG, Beardsley GP (2001). Human 5-aminoimidazole-4-carboxamide ribonucleotide transformylase/inosine 5′-monophosphate cyclohydrolase. A bifunctional protein requiring dimerization for transformylase activity but not for cyclohydrolase activity. J Biol Chem.

[19] Racanelli AC, Rothbart SB, Heyer CL, Moran RG (2009). Therapeutics by cytotoxic metabolite accumulation: pemetrexed causes ZMP accumulation, AMPK activation, and mammalian target of rapamycin inhibition. Cancer Res.

[20] Rothbart SB, Racanelli AC, Moran RG (2010). Pemetrexed indirectly activates the metabolic kinase AMPK in human carcinomas. Cancer Res.

[21] Park JA, Shin HY. ATIC gene polymorphism and histologic response to chemotherapy in pediatric osteosarcoma. J Pediatr Hematol Oncol. 2017; 10.1097/MPH.0000000000000802. 10.1097/MPH.0000000000000802 28267080

[22] Rattan R, Giri S, Singh AK, Singh I (2005). 5-Aminoimidazole-4-carboxamide-1-beta-D-ribofuranoside inhibits cancer cell proliferation in vitro and in vivo via AMP-activated protein kinase. J Biol Chem.

[23] Van Den Neste E, Van den Berghe G, Bontemps F (2010). AICA-riboside (acadesine), an activator of AMP-activated protein kinase with potential for application in hematologic malignancies. Expert Opin Inv Drug.

[24] Sengupta TK, Leclerc GM, Hsieh-Kinser TT, Leclerc GJ, Singh I, Barredo JC (2007). Cytotoxic effect of 5-aminoimidazole-4-carboxamide-1-beta-4-ribofuranoside (AICAR) on childhood acute lymphoblastic leukemia (ALL) cells: implication for targeted therapy. Mol Cancer.

[25] Memmott RM, Mercado JR, Maier CR, Kawabata S, Fox SD, Dennis PA (2010). Metformin prevents tobacco carcinogen--induced lung tumorigenesis. Cancer Prev Res.

[26] Huang X, Wullschleger S, Shpiro N (2008). Important role of the LKB1-AMPK pathway in suppressing tumorigenesis in PTEN-deficient mice. Biochem J.

[27] Asby DJ, Cuda F, Beyaert M, Houghton FD, Cagampang FR, Tavassoli AAMPK (2015). Activation via modulation of de novo purine biosynthesis with an inhibitor of ATIC Homodimerization. Chem Biol.

[28] Natsumeda Y, Ikegami T, Olah E, Weber G (1989). Significance of purine salvage in circumventing the action of antimetabolites in rat hepatoma cells. Cancer Res.

[29] Obajimi O, Keen JC, Melera PW (2009). Inhibition of de novo purine synthesis in human prostate cells results in ATP depletion, AMPK activation and induces senescence. Prostate.

[30] Pedley AM, Benkovic SJA (2017). New view into the regulation of purine metabolism: the Purinosome. Trends Biochem Sci.

