# Supplementary material for: Bifunctional enzyme ATIC promotes propagation of hepatocellular carcinoma by regulating AMPK-mTOR-S6 K1 signaling
Source: Cell Commun Signal. 2017 Dec 16;15:52. doi: 10.1186/s12964-017-0208-8 (PMC5732395; doi:10.1186/s12964-017-0208-8)
Supplement: Supplementary file 1 — Supplementary Materials. (DOCX 818 kb) [file 12964_2017_208_MOESM1_ESM.docx]

Supplementary Materials for

**Bifunctional Enzyme ATIC Promotes Propagation of Hepatocellular Carcinoma by Regulating AMPK-mTOR-S6K1 Signaling**

Minjing Li, Changzhu Jin, Maolei Xu, Ling Zhou, Defang Li, Yancun Yin *

*To whom correspondence should be addressed. E-mail: yinyc1985@126.com

This file includes: Supplementary Figures 1-4





**Sup. Fig 1.** **TNM stage and histologic grade postively correlates with overall and disease-free survival of HCC patients.** Clinical data of HCC patients from the Liver Hepatocellular Carcinoma (TCGA, Provisional) database (<http://www.cbioportal.org/>). Patients were separated into two or three groups based on person gender, diagnosis age, TNM stage, histologic grade and % overall survival or disease-free survival vs. time was plotted.


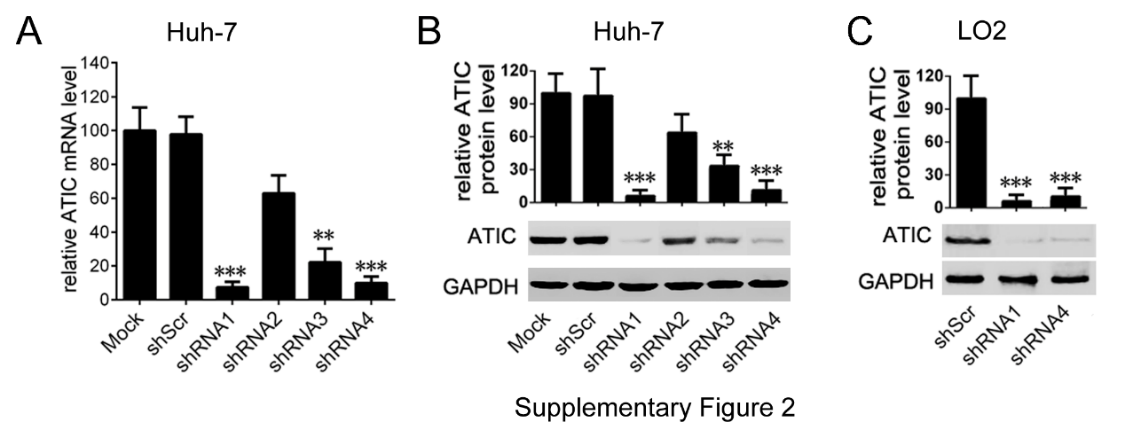


**Sup. Fig 2.** **Transient knockdown expression of ATIC in Huh-7 and LO2 cells. A,** Huh-7 cells were transfected with indicated shRNA for 24 h followed by real-time-PCR analysis of ATIC transcription. N=3, **, p<0.005, ***, p<0.001, compared to shScr and mock group. **B,** Huh-7 cells were transfected with indicated shRNA for 72 h followed by western blot analysis of ATIC expression. The result of densitometric analysis was plotted after normalization to GAPDH. **C,** LO2 cells were infected with indicated shRNA for 72 h, followed by western blot analysis of ATIC expression. The result of densitometric analysis was plotted after normalization to GAPDH.


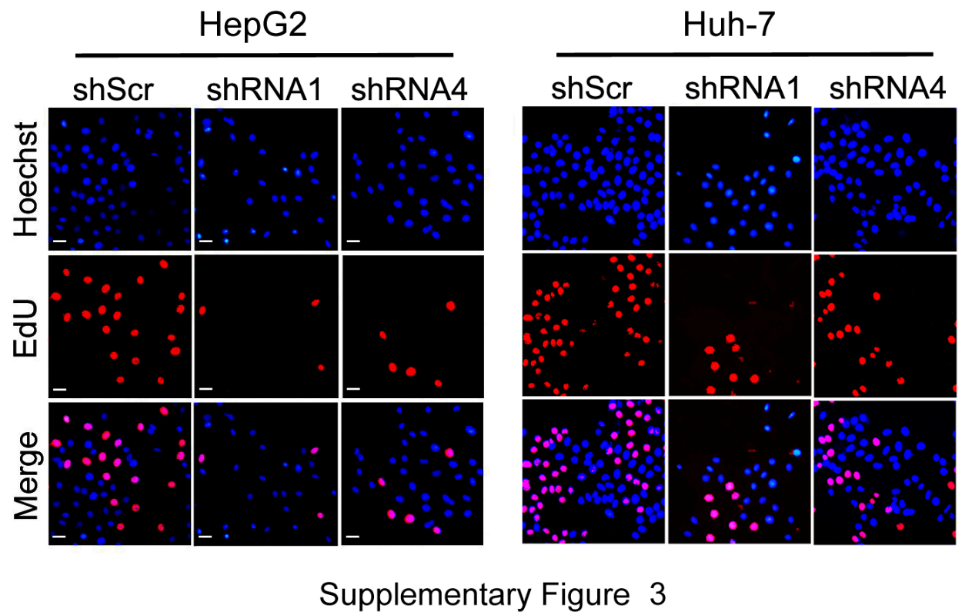


**Sup. Fig 3. Knockdown expression of ATIC inhibited proliferation of HCC cells.** As shown in Fig. 4F, HepG2 or Huh-7 cells were transfected with indicated shRNAs for 72 h followed by Hoechst and EdU labeling analysis proliferation. Representative images were shown. EdU-labeled indicated proliferation cells (Red). Bars, 10 μm.





**Sup. Fig 4. The efficiency of AMPK knockdown.** As shown in Fig. 6H-J, HepG2 cells were infected with ATIC shRNA for 24 h, then were infected with AMPK shRNA for 48 h. The efficiency of AMPK knockdown was determined by western blot.
